# Supplementary material for: A novel user clustering and efficient resource allocation in non-orthogonal mutliple access for IoT networks
Source: PLoS One. 2024 Sep 9;19(9):e0309695. doi: 10.1371/journal.pone.0309695 (PMC11383250; doi:10.1371/journal.pone.0309695)
Supplement: Attachment — Submitted filename: Author Responce Sheet.pdf [file pone.0309695.s001.pdf]

A Novel User Clustering and Efficient Resource Allocation in Non-Orthogonal Mutliple Access for IoT Networks

Syed Muhammad Hamedoon, Jawwad Nasar Chattha, Muhammad Bilal

August 9, 2024

Author Response of Reviewer 1

Reviewer 1: The authors presented an iterative optimization process, starting with user clustering and then moving to power allocation. The proposed user clustering technique used partial brute force search (P-BFS), whole power allocation was performed using the Lagrangian multiplier method with Karush-Kuhn-Tucker (KKT). The article is well written; however, the following issues must be addressed:

Response:

Thank you for reviewing the manuscript and for providing constructive feedback. We appreciate the time and effort you have dedicated to reviewing our work.

Comment No.01

Huge use of constructions in first person. Examples: WE proposed..., WE calculate..., OUR work (abstract and introduction). Rewrite.

Responce:

We have revised the manuscript to reduce the use of first-person constructions. Below are the changes made in the **abstract** and **introduction** sections, highlighted in blue for your reference. These changes have been implemented throughout the manuscript to improve readability and adhere to the feedback provided.

In this paper, user clustering and power allocation challenges in the downlink of a multi-carrier NOMA system are investigated, with sum rate as the optimization objective. The paper presents an iterative optimization process, starting with user clustering followed by power allocation of the users.

- A low-complexity user clustering approach is proposed to optimize the performance of IoT-based NOMA networks.
- A DNN-based proposed partial brute force search (P-BFS) resource allocation scheme is presented further to reduce the complexity of the proposed P-BFS method.
- Optimal power allocation for the IoT devices in the respective clusters is calculated according to the QoS requirements. This power allocation is based on KKT conditions to achieve high energy efficiency in downlink PD-NOMA.
- The results of the proposed and DNN-based P-BFS resource allocation schemes are compared with those of fixed and optimal power allocations.

The findings clearly show an improved system sum rate using optimal power allocation. The performance of the proposed P-BFS-based user clustering techniques is very close to that of the exhaustive BFS and superior to the other channel gain-based user clustering technique.

Comment No.02

The references are extensive and relevant, but there is no clear explanation of how the literature review was conducted. A brief section detailing the bibliographic research methodology and the scientific bases used would enhance the transparency and credibility of the literature review process. References such as "An efficient method for resource allocation and user pairing in downlink non-orthogonal multiple access system" and " Filter orthogonal frequency-division multiplexing scheme based on polar code in underwater acoustic communication with non-Gaussian distribution noise" could be added.

Response:

Thank you for reviewing the manuscript and providing detailed comments that need to be addressed. We have enhanced the explanations within the existing literature review section to provide more detailed insights into related research literature. This includes a more thorough discussion of the methodologies, findings, and contributions of the cited studies to the field of NOMA resource allocation and user clustering.

Furthermore, the following references [28],[29] suggested by the reviewer have been added to the manuscript:

In [18], a user pairing scheme based on Stackelberg game theory is proposed to maximize the sum rate and ensure QoS requirements. First, the solution in the case of two users, where the possibility of dividing the transmit power between the two users is shown or only between the user with the better channel condition is also provided. An iterative approach is then described based on the developed algorithm for a related two-user scenario

An iterative technique is proposed in [21] to optimize power allocation and energy efficiency in mmWave massive MIMO-NOMA systems. The precoding scheme employed is a hybrid one which however takes into consideration the number of RF chains available at the base station. Based on this, a novel iterative power allocation has been developed to achieve an optimum power in order to enhance the energy efficiency.

The performance of NOMA in mmWave communication is evaluated in terms of spectral efficiency (SE) and energy efficiency (EE) discussed in [24]. Firstly, a user grouping approach is provided based on channel

correlations of a user. Further, to sum up, a new hybrid beamforming and power allocation problem are introduced with the objective of achieving maximum sum rate while satisfying the minimum rate requirement for each user. The proposed solution for the mmWave-NOMA for user grouping, joint power allocation, and hybrid beamforming exploits all the benefits to achieve higher sum rate and energy efficiency in comparison to the benchmark scheme but with a higher computational complexity. In [25] author considers a NOMA heterogeneous network (HetNet) in mmWave communications, comprising macro-cell and small cell tiers connected via wireless backhaul. A user grouping algorithm is proposed to significantly simplify the clustering process, with each cluster consisting of highly correlated users to suppress inter-cluster interference

A novel method called NOMA-PKKT-HNG introduced in [28] to address power allocation and user pairing challenges in downlink NOMA systems, aiming to maximize the system's total rate. The non-convex constrained problem was solved by constructing optimal power allocation's closed-form solutions using Karush-Kuhn-Tucker (PKKT) conditions. In [29], the underwater communication challenges are limited bandwidth, low data rate, propagation delay, and high bit error rate (BER). The coding scheme improved the rate of error-corrected data while effectively reducing the Bit Error Rate (BER) in the communication system.

Comment No.03

It is not enough to explore the existing works without highlighting their limitations.

Response:

In response to your comment, we have added related literature in the introduction to specifically discuss the limitations of existing user clustering techniques compared to our proposed method. This addition is highlighted blue in the revised manuscript. The complexity of traditional exhaustive BFS is high and takes more time as the input size grows compared to the proposed P-BFS. However, the proposed adaptive user clustering (AUC) approach in [7] is of significant importance as it searches through all possible combinations to find the best clusters, which can be computationally intensive. Similar related work in [8] relies on UC datasets obtained from the traditional Brute-Force search (BF-S) method, which is computationally intensive. The heuristic channel gain-based user clustering is proposed in [9],[10] which is simpler and faster than exhaustive search methods, it often sacrifices optimality, flexibility, and scalability, resulting in less efficient resource allocation and lower overall network performance.

Comment No.04

It is recommended that all symbols used in the article be compiled into a table for easy reference.

Response:

We have compiled all the symbols and notations used in our manuscript into a **Table 1** for easy reference. This table has been added to the revised manuscript also.

Comment No.05

The proposed solution has not been compared with existing solutions to demonstrate its superiority.

Response:

We acknowledge the importance of comparing our proposed solution with existing methods to demonstrate its superiority. In the revised manuscript, we have included a comparative analysis to highlight the advantages of our approach.

Our research demonstrates the superiority of our proposed solution in terms of **sum rate** and **computational complexity**. Through extensive simulations, we measured the effectiveness of our user clustering and resource allocation procedure. We introduced a novel approach that simplifies the Partial Brute Force Search (P-BFS) method by employing a Deep Neural Network (DNN) model. This integration provides sum rates comparable to exhaustive search (B-FS) but with lower computational complexity. The clustering techniques comparison of sum rate performance is shown in **Fig 10**. The P-BFS method is significantly faster than exhaustive search, making it suitable for real-time IoT networks. This method ensures that the constraint set by the available power is met with the goal of attaining the closest to optimal performance as compared to the overall performance of the proposed user clustering and resource distribution plan making it highly effective thus making it superior to others. Our approach outperforms other heuristic methods based on channel gain, offering substantial improvements in user clustering and resource assignment in NOMA-assisted IoT networks as shown in **Fig 10**.

The below text in blue color is added in **Section 3.1** of the manuscript provides more detail for the superiority of the proposed P-BFS user clustering algorithm in-terms of low complexity

$$B_k = \sum_{k=0}^{K-1} \binom{K-1}{k} B_k \tag{10}$$

The set of 2 user pair possible combinations is calculated by using Eq 11

Table 1: List of notations and mathematical symbols

Notation	Description
n	Number of IoT devices
j	Cluster number from set { 1, 2, ..., c}
i	Set of user from {1,2,. . . ,n}
B	Total system bandwidth
P_i^j	Transmit power of the i-th user in cluster j
ω_j	Resource block of the j th cluster
U_{ω_j}	User share resource block in cluster j
$P_{\max}$	Maximum transmission power of the base station
h_i^j	Channel gain of the i-th user inside j cluster
σ_n^2	Variance of AWGN
P_c	Circuit Power
R_i^j	Rate of i-th user in j-th cluster
$R_{\min}$	Threshold to guarantee minimum data rate transmission requirement
ϵ	The minimum power gap for successful SIC
U_{ω_j}	Number of IoT users sharing resource block j
B_K	Bell number to partition K number of users
V_k^j	Partial partition k for cluster j
R_k^j	Rate for each partial partition for cluster j
p_b	Power is allocated to each resource block
b	Bandwidth of each resource block
L	Lagrange function
λ_i, μ_i, π_i	Lagrange variables
Z	Iteration index
γ_i^j	Signal-to-interference-plus-noise ratio (SINR) of i- th user from cluster j
n^j	Number of user belongs to cluster j
I	Number of input layer nodes
H	Number of hidden layer nodes
V	Number of output layer nodes
x	Input vector for DNN based P-BFS
Z	Output vector for DNN based P-BFS
p	Hidden layer index
N	Number of hidden layer nodes
W^p	Weight matrix that links $(p-1)$ to p-th layer
f^p	Activation function of the p-th layer
b^q	Bias vector for the p th layer
a^p	Output vector of hidden layer
Z_k^*	Predicted sum rate at the output of DNN based P-BFS
H_α	Cost function

$$\text{Possible number of } k \text{ user pairs} = \frac{(2k)!}{2^k.k!} \tag{11}$$

where $n = 2k$, the number of ways to form k pairs. When $n = 10$ the total possible combination for 2 user pair is 945 this reduces the overall combinations as compare to Eq 10.

The time complexity of traditional BFS scheme is $O(n!)$ which exponentially grows for the large number of n. The time complexity of the proposed user clustering based on P-BFS given in Algorithm 1 is $O(n.2^n)$. Therefore, the time complexity of traditional BFS will higher and take more time as the input size grows compared to a proposed P-BFS Algorithm 1.

The possible number of user pairs for $n = 18$ using traditional BFS method from Eq 11 is $34,650$ which significantly larger in numbers as compare to proposed P-BFS Algorithm 1 which creates 2 clusters and the number of partial partition in each cluster is 945 and 105. The proposed P-BFS Algorithm 1 select the partition for each cluster which returns higher sum rate and neglect the other partial partitions V_k as shown in **Fig 2.**

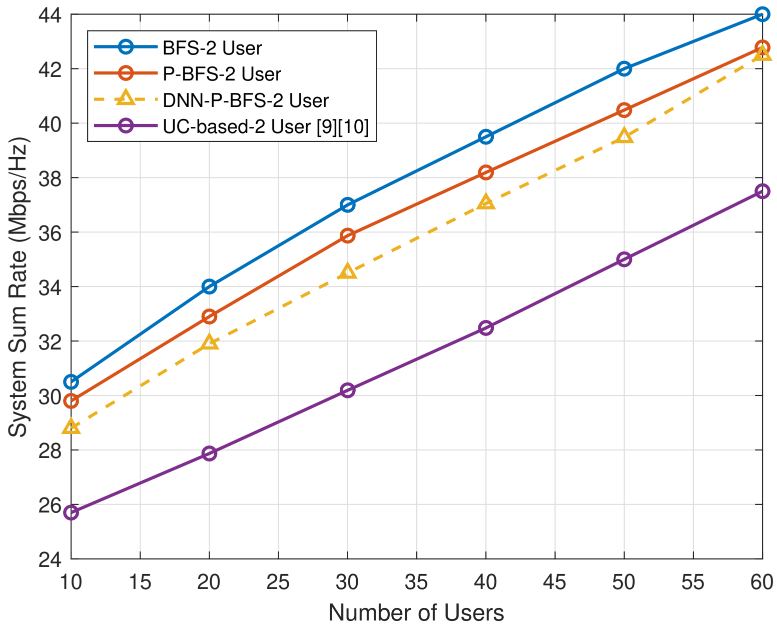

Figure 10: User Clustering Sum Rate Comparison

The below text in blue color is added in **Section 4: Simulation Results** of the manuscript to provide more detail for the superiority of the proposed

P-BFS user clustering algorithm in-terms of system sum rate improvement.

The proposed P-BFS-based user clustering is compared with an exhaustive BFS user clustering scheme in terms of system sum rate. The traditional exhaustive BFS is considered as a benchmark for user clustering data set in [7] and [8]. The proposed P-BFS-based scheme consumes low computational cost in terms of complexity, and the sum rate performance of P-BFS is very close to the traditional exhaustive BFS method, as shown in Fig10. We also compare the proposed P-BFS user clustering scheme with other heuristic channel-based user clustering (UC) proposed in [9],[10]. In user clustering, pairs of users are selected based on the highest difference in channel gains between two users, starting with the highest channel gain of the first user paired with the lowest channel gain of the last user, and so on. The same simulation and system parameters are chosen from Table 3 for user clustering comparison. Our approach outperforms other user clustering techniques as shown in Fig 10, offering substantial improvements in user clustering and resource assignment in NOMA-assisted IoT networks.

Thank you once again for your valuable feedback. The revised manuscript has been uploaded for your review.

Author Response of Reviewer 2

Reviewer 2: The authors introduced an iterative optimization process, starting with user clustering followed by power allocation. Although the article is well-written, the following issues need to be addressed:

Response:

Thank you for reviewing the manuscript and constructive feedback on my document. We appreciate the time and effort you have dedicated to reviewing our work.

Comment No.01

The assumptions made in the modeling, such as ideal propagation conditions, might not accurately reflect real-world scenarios. A discussion on how these assumptions impact the generalizability of the findings would be beneficial.

Response:

We have addressed this by adding the following text in the system model. It is assumed that the BS has complete information about the CSI for its associated users. The channels between the BS and IoT users are modeled using Rayleigh fading as in [14].

Comment No. 02

The paper does not provide a detailed comparison of the proposed methods with existing user clustering and resource allocation techniques, making it difficult to evaluate the relative superiority or efficiency of the new approach.

Response:

We acknowledge the importance of comparing our proposed solution with existing methods to demonstrate its superiority. In the revised manuscript, we have included a comparative analysis to highlight the advantages of our approach.

Our research demonstrates the superiority of our proposed solution in terms of **sum rate** and **computational complexity**. Through extensive simulations, we measured the effectiveness of our user clustering and resource allocation procedure. We introduced a novel approach that simplifies the Partial Brute Force Search (P-BFS) method by employing a Deep Neural Network (DNN) model. This integration provides sum rates comparable to exhaustive search (B-FS) but with lower computational complexity. The clustering techniques comparison of sum rate performance is shown in **Fig 10**. The P-BFS method is significantly faster than exhaustive search, making it suitable for real-time IoT networks. This method ensures that the constraint set by the available power is met with the goal of attaining the closest to

optimal performance as compared to the overall performance of the proposed user clustering and resource distribution plan making it highly effective thus making it superior to others. Our approach outperforms other heuristic methods based on channel gain, offering substantial improvements in user clustering and resource assignment in NOMA-assisted IoT networks as shown in **Fig 10**.

The below text in blue color is added in **Section 3.1** of the manuscript to provide more detail for the superiority of the proposed P-BFS user clustering algorithm in-terms of low complexity

$$B_K = \sum_{k=0}^{K-1} \binom{K-1}{k} B_k \tag{10}$$

The set of 2 user pair possible combinations is calculated by using Eq 11

$$\text{Possible number of } k \text{ user pairs} = \frac{(2k)!}{2^k.k!} \tag{11}$$

where $n = 2k$, the number of ways to form k pairs. When $n = 10$ the total possible combination for 2 user pair is 945 this reduces the overall combinations as compare to Eq 10.

The time complexity of traditional BFS scheme is $O(n!)$ which exponentially grows for the large number of n. The time complexity of the proposed user clustering based on P-BFS given in Algorithm 1 is $O(n.2^n)$. Therefore, the time complexity of traditional BFS will be much less efficient and take more time as the input size grows compared to a proposed P-BFS Algorithm 1

The possible number of user pairs for $n = 18$ using traditional BFS method from Eq 11 is $34,650$ which significantly larger in numbers as compare to proposed P-BFS Algorithm 1 which creates 2 clusters and the number of partial partition in each cluster is 945 and 105. The proposed P-BFS Algorithm 1 select the partition for each cluster which returns higher sum rate and neglect the other partial partitions V_k as shown in **Fig 2.**

The below text in blue color is added in **Section 4: Simulation Results** main manuscript to provide more detail for the superiority of the proposed P-BFS user clustering algorithm in-terms of system sum rate improvement

The proposed P-BFS-based user clustering is compared with an exhaustive BFS user clustering scheme in terms of system sum rate. The traditional exhaustive BFS is considered as a benchmark for user clustering data set in [7] and [8] The proposed P-BFS-based scheme consumes low computational cost in terms of complexity, and computational sum rate performance is very close to the traditional exhaustive BFS method, as shown in Fig10. We also

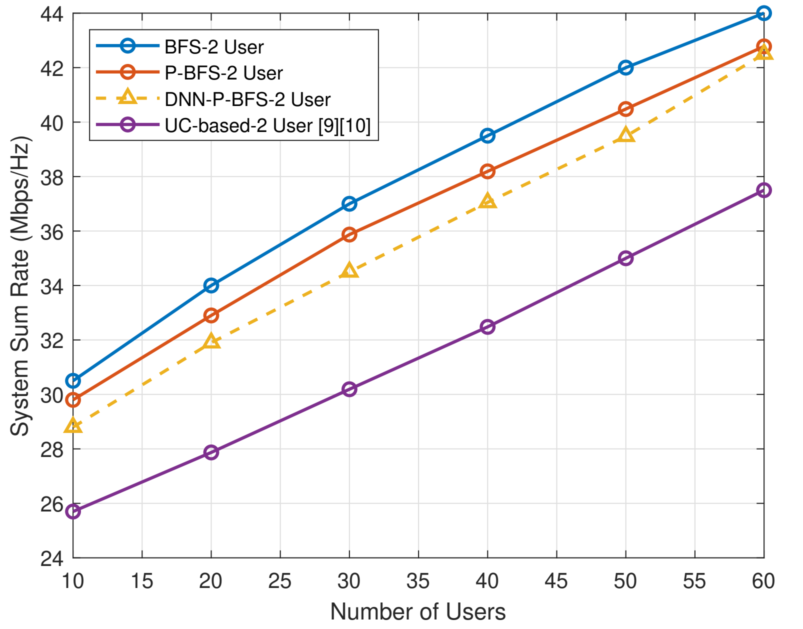

Figure 10: User Clustering Sum Rate Comparison

compare the proposed P-BFS user clustering scheme with other heuristic channel-based user clustering (UC) proposed in [9],[10]. In user clustering, pairs of users are selected based on the highest difference in channel gains between two users, starting with the highest channel gain of the first user paired with the lowest channel gain of the last user, and so on. The same simulation and system parameters are chosen from Table 2 for user clustering comparison. Our approach outperforms other user clustering techniques as shown in Fig 10, offering substantial improvements in user clustering and resource assignment in NOMA-assisted IoT networks.

Comment No.03

It is advised to compile all symbols used throughout the article into a table to facilitate easy reference.

Response:

We have compiled all the symbols and notations used in our manuscript into a **Table 1** for easy reference. This table has been added to the revised manuscript also.

Thank you once again for your valuable feedback. The revised manuscript has been uploaded for your review.

Table 1: List of notations and mathematical symbols

Notation	Description
n	Number of IoT devices
j	Cluster number from set { 1, 2, ..., c}
i	Set of user from {1,2,. . . ,n}
B	Total system bandwidth
P_i^j	Transmit power of the i-th user in cluster j
ω_j	Resource block of the j th cluster
U_{ω_j}	User share resource block in cluster j
$P_{\max}$	Maximum transmission power of the base station
h_i^j	Channel gain of the i-th user inside j cluster
σ_n^2	Variance of AWGN
P_c	Circuit Power
R_i^j	Rate of i-th user in j-th cluster
$R_{\min}$	Threshold to guarantee minimum data rate transmission requirement
ϵ	The minimum power gap for successful SIC
U_{ω_j}	Number of IoT users sharing resource block j
B_K	Bell number to partition K number of users
V_k^j	Partial partition k for cluster j
R_k^j	Rate for each partial partition for cluster j
p_b	Power is allocated to each resource block
b	Bandwidth of each resource block
L	Lagrange function
λ_i, μ_i, π_i	Lagrange variables
Z	Iteration index
γ_i^j	Signal-to-interference-plus-noise ratio (SINR) of i- th user from cluster j
n^j	Number of user belongs to cluster j
I	Number of input layer nodes
H	Number of hidden layer nodes
V	Number of output layer nodes
x	Input vector for DNN based P-BFS
Z	Output vector for DNN based P-BFS
p	Hidden layer index
N	Number of hidden layer nodes
W^p	Weight matrix that links $(p-1)$ to p-th layer
f^p	Activation function of the p-th layer
b^q	Bias vector for the p th layer
a^p	Output vector of hidden layer
Z_k^*	Predicted sum rate at the output of DNN based P-BFS
H_α	Cost function
